# Energy-Efficient and Fast MAC Protocol in UAV-Aided Wireless Sensor Networks for Time-Critical Applications

**DOI:** 10.3390/s20092635

**Published:** 2020-05-05

**Authors:** Sabitri Poudel, Sangman Moh

**Affiliations:** Department of Computer Engineering, Chosun University, 309 Pilmun-daero, Dong-gu, Gwangju 61452, Korea; poudelsabitri32@gmail.com

**Keywords:** wireless sensor network, unmanned aerial vehicle, medium access control, network protocol, energy efficiency, delay minimization, network lifetime

## Abstract

Unmanned aerial vehicle (UAV)-aided wireless sensor networks (UWSNs) can be effectively used for time-critical sensing applications. UAVs can be used to collect the sensed data from sensors and transfer them to a base station. The real-time transfer of data is highly desired in the time-critical applications. However, the medium access control (MAC) protocols designed for UWSNs so far are primarily focused on the efficient use of UAVs to collect data in the sensing areas. In this paper, we propose an energy-efficient and fast MAC (EF-MAC) protocol in UWSNs for time-critical sensing applications. EF-MAC adopts carrier sense multiple access (CSMA) for the registration of sensor nodes with a UAV and time division multiple access (TDMA) with variable slot time for the transmission of collected data. The UAV is equipped with two transceivers to minimize both energy consumption and delay in air-to-ground communication. The energy consumption and delay are formally analyzed and the performance of EF-MAC is evaluated via extensive simulation. The simulation results show that the proposed EF-MAC outperforms the conventional MAC protocols in terms of energy efficiency and communication delay.

## 1. Introduction

Wireless sensor networks (WSNs) have become popular in a wide range of applications aimed at the modernization and betterment of our society [1,2]. The applicability of WSNs has been enhanced by advances in the technology and availability of small, inexpensive, and smart sensors resulting in cost effective and easily deployable WSNs. Sensor nodes are designed to perform high-level information processing tasks such as detection [3], classification [4], tracking [5], monitoring, and surveillance [6]. In WSN applications, sensors are usually deployed such that they are directly or indirectly connected with a base station (BS) which will receive the sensing information. In most WSNs, sensor nodes are grouped into clusters. Every sensor node sends the sensed data to its cluster head (CH), and the CH aggregates the data received from its members and then sends the aggregated data to the BS.

However, fully connected network of sensor nodes may not be established in remote areas. In addition, we cannot rely on previously established infrastructures in harsh environments (i.e., wars and disasters) because there is no guarantee that the built network will remain operational as discussed in [7]. Moreover, energy consumption is significantly high and unequal one if sensor nodes need to transfer the sensed information to BS through multi-hop communication. Mission and time-critical WSN applications are defined as target applications that demand data delivery in the bounds of time and reliability. A vast number of time-critical applications can be found in the literature [8,9], which require high throughput with minimum delay. Furthermore, energy consumption remains a design concern, as a reasonably long network lifetime is always desirable. Thus, employing a mobile sink for data gathering is an option to fortify such applications.

Unmanned aerial vehicles (UAVs), on the other hand, have received tremendous interests in both research community as well as civil applications such as surveillance and reconnaissance operations [10]. With the recent advancement in wireless networking technologies, the use of UAV has been incorporated in many applications. UAV is capable of sensing or collecting data from a wider area within a faster period. Different routing and clustering approaches for UAVs are studied in [11,12]. Other promising features of UAV include small size, easy deployment, low maintenance cost, improved safety for humans, environmental flexibility, and high maneuverability [13]. Thus, it is suitable to deploy the UAV for disaster management operations where the safety of human and time-sensitive data collection is of prime concern [14,15]. Correspondingly, the increasingly rapid incorporation of UAVs in other wireless technologies has been studied.

One of such integrations is a UAV-aided WSN (UWSN). In UWSNs, UAVs can be effectively utilized for several different purposes such as deployment of sensor nodes [16], extension of communication range [17], and wireless power transfer in WSNs [18], data gathering from ground sensor nodes [19], maintaining connectivity [20], and localization of sensor nodes [21]. UWSNs are different from mobile WSNs where sensor nodes or sink may move [22]. In UWSNs, UAVs are used to receive data from sensor nodes directly, which greatly reduces the communication between sensor nodes and BS and saves the energy required for listening to neighbors. Another advantage of UWSNs is the presence of a free space between sensor fields and UAVs. The free space reduces the chances of signal decaying, which is very prominent in sensor-to-BS communication. In addition, UAVs can detect weak signals because they are equipped with high-level signal processing units and multiple antennas.

For guaranteeing high network throughput and low energy consumption in UWSNs, several issues must be addressed in the medium access control (MAC) layer. Existing MAC protocols designed for WSNs or UAVs do not perform well in UWSNs because of the high mobility of UAVs and the limited contact time between UAVs and sensor nodes. Even though there are some similarities between UWSNs and vehicular ad hoc networks (VANETs), the MAC protocols utilized in VANETs are also unsuitable for UWSNs owing to the densely employed sensor nodes, which results in collisions while communicating with UAVs. Due to the rapid mobility of UAVs, the dynamic change of network topology, and the constraints of operation time and energy in UWSNs, designing an efficient MAC protocol that addresses abovementioned issues is a challenging task. All the extant MAC protocols for UWSNs are compared and studied in [23].

**Figure 1:**
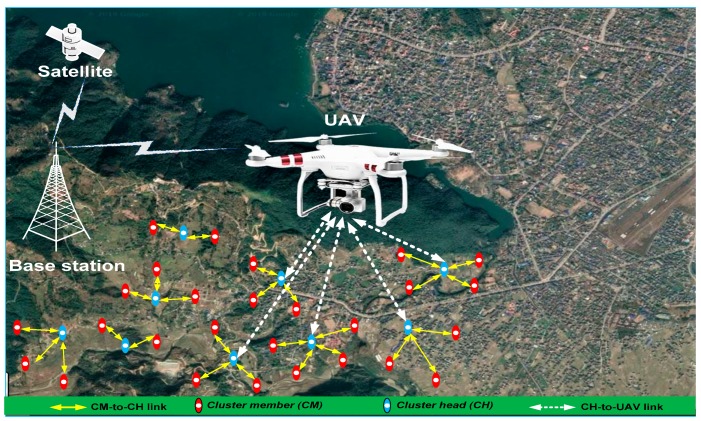
Application scenario of unmanned aerial vehicle (UAV)-aided data gathering in wireless sensor networks (WSN) for mission and time-critical applications.

In this paper, we propose energy-efficient and fast MAC (EF-MAC) in UWSNs for time-critical applications. The application scenario of the proposed protocols is depicted in Figure 1. In the figure, sensor nodes are grouped into clusters and each CH collects data from its cluster members. Every CH then transmits the aggregated data to the UAV when it receives beacon signals from UAV.

### 1.1. Contribution of the Study

In this paper, it is assumed that sensor nodes are clustered and cluster heads are responsible for communicating with the UAV. As mentioned earlier, many clustering schemes have been developed in WSNs for many years. The proposed EF-MAC significantly decreases both energy consumption and delay in air-to-ground communication. In our work, UAV’s energy consumption is not emphasized because UAV is assumed to have enough energy for data gathering operation and gets charged once it returns to BS. The major contributions of our work are as follows:

- We present a hybrid MAC protocol for time-critical sensing applications in emergency and disaster environments by incorporating two transceivers in a UAV. The two transceivers operate concurrently for minimizing both energy consumption and delay in air-to-ground communication. It should be noted that sensor nodes are clustered and only CHs communicate with UAV.
- The proposed EF-MAC uses carrier sense multiple access (CSMA) for registration process. For data transmission, on the other hand, time division multiple accesses (TDMA) with variable slot time is used. As a result, proper synchronization is done before exchanging data packets.
- The prioritization of registered sensor nodes is carried out before scheduling process. Priority is assigned on the basis of contact duration time (CDT), packet size, and residual energy. If a CH goes out of communication range of UAV, it is discarded for scheduling.
- The EF-MAC protocol is mathematically analyzed with respect to delay and energy consumption. Markov chain-based modeling is presented for delay and energy analysis of CHs at different stages of communication.
- Our performance study shows that the proposed EF-MAC outperforms the existing CSMA with collision avoidance (CSMA/CA) and hybrid MAC protocol (HP-MAC) in terms of delay, energy usage, throughput, network lifetime, and fairness.

### 1.2. Outline of the Paper

The rest of this paper is organized as follows. Related works are summarized in brief in the following section. The system model for our study is described in Section 3. The proposed EF-MAC is presented in detail along with the devised algorithms in Section 4. The delays and energy consumption of EF-MAC are analyzed and discussed in Section 5. The performance of the proposed EF-MAC is evaluated through a computer simulation and compared with conventional MAC protocols in Section 6. Finally, the paper is concluded in Section 7.

## 2. Related Works

UWSNs applications are attracting a lot of attention from industry and academia. Nowadays, incorporating UAVs in WSNs for data disseminating and gathering has been a common trend. However, proper coordination between UAV and WSN is prerequisite of UWSN networks. Hence, in UWSNs, MAC needs to be designed for obtaining high network throughput within low energy usage. Additionally, due to the movement of the UAV, there is a short time interval during which data can be transmitted from the sensor to the UAV. Having said that, we can find that immense research is going on for the enhancement of MAC protocols for UWSNs in the literature. It has been shown that IEEE 802.11 CSMA MAC is not suitable for the UWSN system although it has been widely used in wireless communications. This is due to the following reasons: it suffers from longer contention delay during UAV-to-CHs communication and it becomes worse in the dense network. Another issue faced by CSMA/CA MAC is hidden terminal effect and the exchange of request to send and clear to send (RTS/CTS) adds overhead and results in delay. Another conventional MAC protocol i.e., TDMA eliminates the issues of collisions and re-transmission by pre-allocating the transmission resources to the nodes in the network but it requires information of the network before scheduling nodes.

Adaptive-opportunistic Aloha has been proposed in [24] which being based on cross layer design considers the uniformity of data collection, energy consumption, and transmission efficiency. Code division multiple access (CDMA) is considered for physical transmission. A novel multiple access scheme for aerial sensor networks employing adaptive antenna arrays with spatial reuse is proposed in [25]. A priority-based optimized frame selection (POFS), a circularly optimized frame selection scheme (COFS), and a priority-based contention window adjustment scheme (PCWAS) with a collision resolution mechanism are introduced in [26].

A prioritized frame selection (PFS) based CDMA MAC (PFSC-MAC) [27,28,29] incorporates both the beacon signal receiving power and the variation trend of this factor to lower down packet error rate (PER) value. Integration of PFS-TDMA (PFST) and FRA schemes (frame based random access) are also presented in the literature [30] with findings such as optimal number of sub groups, data packet size, and altitude of the UAV under an acceptable ratio of PER and the number of sensors in the network.

A novel MAC protocol in UWSNs, called advanced prioritized MAC (AP-MAC) is proposed in [31]. The authors in [32] proposed a cooperative sensing data-collecting framework for UWSNs. Being based on the IEEE 802.11 CSMA/CA MAC, the neighboring nodes can receive and store the overhearing data into their received buffer. The neighboring node can retransmit the overheard data whenever needed, such as in the case, if an original node is not able to transmit its data to the UAV because it gets out of the UAV communication range. HP-MAC, a hybrid MAC protocol, based on the IEEE 802.11 CSMA/CA MAC protocol and TDMA protocol is proposed in [33]. A hybrid protocol that partially adopts the beacon-based IEEE 802.15.4 MAC DR/CDT mechanisms is proposed in [34]. Recently, MAC for UAV-based mission-critical WSNs is proposed in [35]. The authors have focused on designing MAC for efficient communication and coordination between UAVs employed in a mission. However, UAV-to-sensor communication is not considered.

## 3. System Model

### 3.1. Clustering of Sensor Nodes

**Figure 2:**
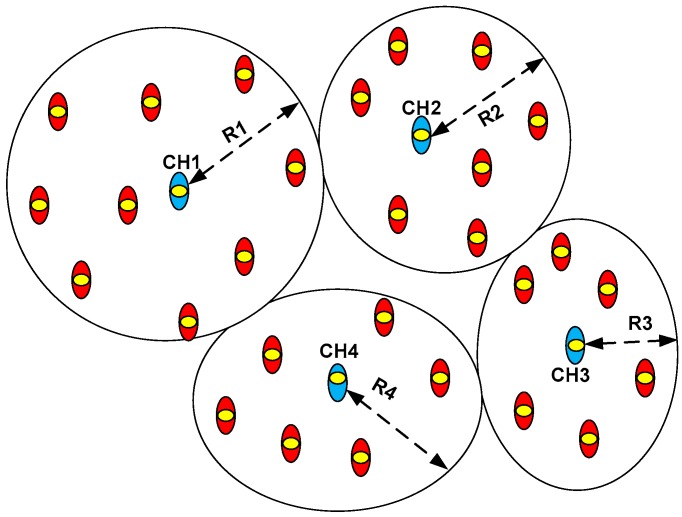
Cluster head (CH) selection in unequal size clustering method as in [37].

It is assumed that homogenous sensor nodes are randomly deployed on the ground. The sensor nodes are clustered using an energy-efficient clustering method as presented in [36,37]. At first, sensor nodes share their head status to all the sensor nodes within its transmission range. The head status includes node identifier, residual energy and location coordinates. The node having the highest energy is elected as CH in that region. Figure 2 clarifies CH selection process where circles represent different competition ranges. If *CH_1_* is selected as CH, no other CH will be selected within its competition range *R_1_*. The selection of CHs is controlled over the network. The competition range for *CH_i_* is determined as in [37], where 0 ≤ *i* ≤ *n* and *n* is the number of CHs. That is,

(1) $$Range_{comp}=1-\alpha \frac{\left(D_{max}-d\left({CH}_{i},{SN}_{j}\right)\right)}{D_{max}-D_{min}}-\beta \left(1-\frac{E_{r}}{E_{max}}\right)R_{max}$$

where *α* and *β* are weights ranging between 0 and 1, *R*_max_ is the maximum transmission range, *E_r_* is the remaining energy of the node, *E*_max_ is the maximum value of energy, *d(CH_i_*, *SN_j_)* is the distance between *CH_i_* and sensor node *SN_j_* (0 ≤ *j* ≤ *N* and *N* is the number of sensor nodes), *D*_max_ and *D*_min_ are the maximum and minimum distance between *SN_j_* and other CHs in the neighborhood.

After a CH is nominated, sensor nodes send join request to the CH. Then, the CH selects its cluster members on the basis of distance and the number of cluster members it can accommodate. If the number of join requests exceeds the cluster size (i.e., the maximum number of nodes in a cluster), the CH rejects the excessive join requests from sensor nodes. The sensor nodes that do not receive the join reply try to join any other CH in their vicinity. CHs are responsible for collecting data from their cluster members before the arrival of the UAV. Data aggregation is carried out to filter redundant data so that the size of data to be transmitted is small. CH selection and cluster formation takes place after the nodes are deployed on ground and before the arrival of UAV. At every round, after UAV-to-CH communication, the remaining energy of CH is compared with the minimum energy required for SN to be CH (i.e., *E*_min_). If the remaining energy of a particular CH is lesser than *E*_min_, a new CH is selected for the next round. Otherwise, the same node continues as CH for the next round as well.

### 3.2. UAV and Antenna Type

We consider a fixed wing UAV for our system, which flies at constant height (*h*) and velocity (*v*) without stopping at any place to gather data from CHs. A fixed wing UAV has the capabilities to be stable during high speed as well as its ability to maneuver during bad conditions. In addition, takeoff and landing are not so complicated due to the lightweight of such UAVs. The flight path of the UAV is predefined such that it travels all the area without stopping to collect data. The UAV is equipped with two transceivers. One of the transceivers is used to broadcast beacon signals and receive registration packets whereas second one is used to send scheduling information and receiving data packets. Information about the antenna bearing is sent by the UAV in beacon signals and scheduling frames. CHs tune to the respective frequencies before transmitting the packets to UAV. UAV uses a directional antenna with flare angle *φ* = 60*°* Directional communication offers many benefits such as extended range of transmission, less delay, and spatial reuse. The communication range of the UAV can be given by *r* = *h tan*(*φ*/2), where *φ* is the angle of the antenna orientation of the UAV. The sensors, which lie on the coverage area of flare angle and receive the beacon signal from the UAV, become active. If sensors do not receive signal or the received signal strength is weak, the sensors go to sleep mode to save energy. In this way, directional antenna limits the coverage of the UAV and overcomes the directional deafness problem.

### 3.3. Localization of Sensor Nodes and UAV

We utilize geographic location information of sensors to improve the accuracy in the localization process. Both UAV and sensor nodes are equipped with global positioning system (GPS) for achieving higher efficiency location information and timing. Location and timing information are used during registration phase by sensor nodes to be registered with the UAV. The UAV uses GPS information during scheduling phase to achieve fairness and efficient use of TDMA slots. In some works [31,38], it is also assumed that only few of the sensor nodes in the network are equipped with local GPS receiver and other sensor nodes can find their position according to GPS empowered sensors to reduce the cost of network. Because our work is highly focused for critical situations, however, the certain amount of additional cost in the network is acceptable. We also assume that the flight path of the UAV covers all the sensors in the field and speed of the UAV is chosen such that it gets in the vicinity of all sensor nodes during its fight time.

### 3.4. Communication Phases

Communication in the proposed EF-MAC system completes in the three steps: i.e., sensor-to-CH communication, CH-to-UAV communication, and the UAV-to-BS communication. For sensor-to-CH communication, after the deployment of sensor nodes in the application area, the sensors are grouped into clusters by using a clustering algorithm. A CH is selected on each cluster and the CH collects data from its cluster members before the arrival of the UAV. Each CH assigns TDMA slots to its cluster members and the cluster members transmit the sensed data to its CH using the allotted slot time. Sensor-to-CH communication is very similar as in WSNs and it has been highly researched in the existing literature. Therefore, we have not given much attention to intra-cluster communication in our work. Then, CHs are also responsible to communicate with UAV and transmit the collected data to the UAV. In addition to this, UAV-to-BS communication occurs using a cellular link. We assume that the UAV can easily transmit the collected data to BS. As a result, energy consumption issue does not exist there. Hence, we have focused our research on designing novel MAC protocol that assures efficient CH-to-UAV communication with minimum delay.

## 4. Energy-Efficient and Fast Medium Access Control

In this section, we have detailed the proposed EF-MAC protocol for emergency environments. Three algorithms are presented in the following subsections, which are EF-MAC at UAV side, EF-MAC at sensor side, and prioritization process, respectively. Algorithms are discussed in association with their operational principles and packet formats.

### 4.1. EF-MAC at UAV Side

In UWSNs, the UAV is responsible for gathering data from the low powered sensors deployed on the ground. Keeping this in mind, channel is divided into four different phases after the arrival of the UAV. Algorithm 1 describes how UAV-MAC functions for efficient UAV-to-CH communication. At first, when the UAV gets into the area of WSN deployment, it announces its presence to the sensor nodes by broadcasting the beacon signals in the current active area by using transceiver *T*_1_. Beacon frame includes information about the UAV such as current position, velocity, transceiver *T*_1_, and time. This phase is known as beaconing phase. After transmitting beacon signals, UAV waits for sensor nodes to send registration frames. This is the second phase i.e., registration phase. Information of CHs is embedded in the registration frame. Third phase i.e., scheduling phase begins after UAV receives registration frames from the sensors in its active area. By using the information of CHs in registration frame, the UAV calculates CDT with every CH in the list, remaining energy of CH and the amount of data to be transmitted. Afterwards, the UAV assigns priority to CHs and then allocates time slots being based on that priority. Size of the time slot is decided considering the data size buffer of CHs. In this way, the channel time is not wasted and ultimately the energy of the network is reduced to great extent. Then, the UAV broadcasts scheduling information by using the transceiver (*T*_2_) and this phase is known as scheduling phase. Finally, the UAV receives data packets from the CHs in the fourth phase i.e., data gathering and transmits it to the BS. CHs use the assigned slots to transfer data so there is no collision during the data gathering process. Thus, EF-MAC significantly reduces the time and energy consumption due to multiple re-transmissions.
**Algorithm 1.** The algorithm is run in the UAV as it gets into the area of a WSN to establish communication with CHs.**Input:** UAV_id*,* location (*x*, *y*, *z*), velocity (*v*), transceiver bearings (*T*_1_ and *T*_2_), and RegisteredCHList = {*Ø*} **Output:** TDMA slots with variable time /*Initialize the network*/ //The flight time, path and velocity of UAV is determined and controlled by the BS. UAV flies on its preplanned path and schedule to gather data from the sensor nodes. 1:   **begin** 2:                  **while** (*UAV is in the area of WSN*) **do**//when UAV reaches the area of sensor nodes deployment 3:                        Send beacon frames//UAV transmits beacon signals to let the sensors know about its presence 4:                        **if** (*receive registration frames from CHs*) **then**//after beaconing process, UAV waits for the registration frames from CHs /*Update*/ 5:                          RegisteredCHList = {*CH_i_*, *CH_j_*, *CH_k_*, …}//UAV maintains a queue to keep record of the registered CHs and it is updated every time UAV receives registration frame from CHs 6:                          Use prioritization process// Prioritization process is described in Section 4.3. 7:                           Schedules CHs on the basis of prioritization process 8:                          Send scheduling frames to CHs //after scheduling the registered CHs, UAV transmits the scheduling frames to CHs 9:                          Receive data frames from CHs//UAV receives data frames from CHs in final phase 10:        **end if** 11:        Remove CH from the RegisteredCHList*/*/after data gathering process, the CH is removed from the list /*Update*///UAV updates its RegisteredCHList so that the CHs that transmitted their data will not be scheduled again 12:     **end while** 13:   **end**

### 4.2. EF-MAC at Sensor Side

**Figure 3:**
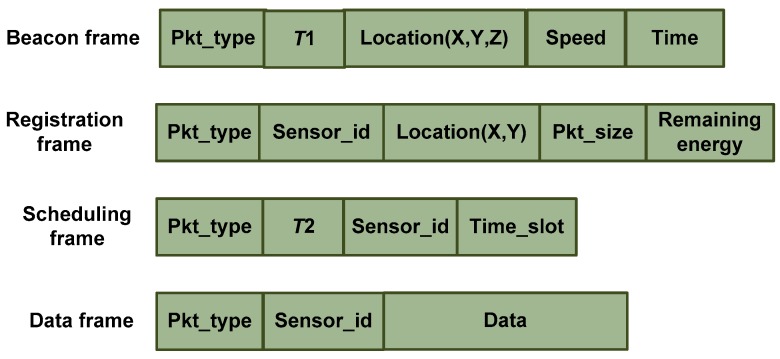
Format of packets for energy-efficient and fast medium access control(EF-MAC).

Owing to the mobility of the UAV, CHs need to transmit their data to the UAV before it leaves the coverage area of the UAV. Hence, there must be an efficient MAC running at WSN to ensure proper scheduling of the sensor nodes. After collecting data from the cluster members, CH sleeps and periodically wakes up to check beacon signals. Once CHs receive beacon signals from the UAV, they get active and immediately contend a channel to send registration frame to the UAV. During this phase, CHs use CSMA MAC protocol and use random back-off in case of collision. The registration frame size is very small compared to the data size and exchange of RTS/CTS is excluded to reduce the network overhead. In addition, the active area is bounded by the smaller beam-width of antenna and sensors are clustered so we assume that there is tolerable contention delay. Registration frame includes the information of CHs such as sensor_id, the position information *(X_s_*, *Y_s_)* remaining energy and data buffer size. This whole process is carried out on the registration phase. Before transmitting registration frame, CHs use the location and speed information of the UAV, which are received in beacon signals, to calculate the current position of the UAV as:

(2) $$X_{C}=X_{i}+V\times \left(t_{c}-t_{i}\right)$$

(3) $$Y_{C}=Y_{i}+V\times \left(t_{c}-t_{i}\right)$$

and

(4) $$Z_{C}={\;Z}_{i}+V\times \left(t_{c}-t_{i}\right)$$

where the first term (i.e., *X_i_*, *Y_i_*, and *Z_i_)* in every equation gives the current axes coordinates of the UAV, respectively. The second term (i.e., *V* × (*t_c_*−*t_i_*)) denotes the distance covered by the UAV in a certain time frame. We assume that CHs will contend the channel only if they have some data to transfer to the UAV otherwise, they will sleep until next active period to save the energy. Additionally, we assume that CHs aggregate the collected data from its cluster members and drop the redundant data. Hence, the amount of data collected by every CH is not of same size. Therefore, variable-length TDMA is introduced to utilize the channel efficiently. Format of four packets used in the four different phases of UAV-to-CHs communication is shown in Figure 3.

CHs wait for the scheduling frames after sending the registration frame to the UAV. If the CH fails to receive scheduling frame in that particular period due to collision or other network issues then it tries again after random time until it is in the active region of the UAV and does not exceed the retries count limit. CHs receiving the scheduling information will wait for their turn in the frame. Finally, the scheduled CH uses the assigned time slot and transfer data directly to the UAV using *T*_2_ in a data-gathering phase. Hence, the UAV-to-CH link is efficiently utilized regarding time and energy. The MAC implemented at CH is detailed with the help of Algorithm 2.
**Algorithm 2.** The algorithm is run in the ground CHs after the arrival of UAV.**Input:** CH_id*,* location (*x, y*)*,* packet size (*l*)*,* remaining energy*,* and RecieveBCN *=* 0 **Output:** Aggregated variable-sized data packets to transmit to UAV /*Initialize the network*/ //After the sensor nodes are deployed on the ground, they form a cluster and select CH for each group. CH collects data from its cluster members by assigning proper TDMA slots and aggregates the collected data. Redundant data are discarded by CHs during the aggregation process and CHs wait for the UAV’s arrival 1:   **begin** 2:     **if** (*RecieveBCN =* 1) **then** //CHs wake up periodically to check the arrival of UAV. Once CH senses the UAV’s arrival through the beacon signals, it immediately gets active and tries to send registration frames 3:     **while** (CH is in the active region of UAV’s antenna orientation) **do** 4:             CHs get active and contend for registration //All CHs in active area tries to communicate with UAV using contention-based method i.e., CSMA /*Update */ 5:              Send registration frame to UAV //CHs contend the channel to send registration frame to the UAV embedding information about its location, packet size, remaining energy, CH_id 6:                **if** (receives scheduling frames from UAV) **then** /*Update*/ //CHs synchronize its time with UAV 7:                Waits for its turn //CHs check their slot time in scheduling frame and wait 8:                Sends data frames //CHs send all the collected data in their time slot and sleep 9:                  **else** 10:                Resends registration frames //CHs keep trying for registration until maximum retry limit is met. If a CH cannot get registered until the time, then there is loss of data packets from the UAV 11:                **end if** 12:                **for** (each CHs in the UAV’s coverage region) **do`** 13:                Repeat Steps 4 to 11 //this is run in every CHs that receives beacon signals from UAV, is in the coverage area of UAV and if the re-transmission limit is not exceeded. 14:                **end for** 15:       **end while** 16:      **end if** 17:     **end**

### 4.3. Prioritization Process

The principal objective of EF-MAC is to maximize the amount of data collection with minimum delay and reduced energy consumption. Apart from energy and delay minimization, the protocol should also be fair enough to collect data from all the cluster heads. Hence, concerning the fairness of the network priority mechanism is introduced where priority is assigned by the UAV to the registered CHs based on their data buffer size, remaining energy and CDT. In this subsection, we describe the priority assignment mechanism with the help of Algorithm 3. Prioritization process is used by the UAV after receiving the registration frames from CHs and before transmitting the scheduling frames. As explained in Algorithm 1, during the registration phase, information regarding the location, data size, and energy level is embedded and transferred to the UAV by CHs. The UAV records this information in a list (i.e., RegisteredCHList). For all the registration packets received, the UAV calculates the distance between the UAV and CHs (i.e., *d*(*UAV*, *CH*)) and also the coverage range (*r*) by using the information of CHs in the registration packets. Afterwards, using the information calculated, the UAV finds out for how long time it can be in the communication range of the particular CH. This information is stored in CDT. If the value of CDT is too less to establish communication then that CH is discarded for that round. It helps to reduce the packet loss rate due to coverage problem. Additionally, the waste of time and energy due to communication failure is minimized. Then, the UAV uses CDT values and the information of data size and also the remaining energy of the CH to finalize the scheduling process. The CH which has large amount of data to transmit and less amount of remaining energy is scheduled first so that the data loss is minimized due to death of CH. The UAV shares the scheduling information with the registered CHs using *T*_2._
**Algorithm 3.** The prioritization process for EF-MAC before scheduling the CHs.**Input:** list of CH_id {*CH*_1_, *CH*_2_, *CH*_3_, …., *CH_n_*}, location *(x*, *y*), data size (*l*)*,* remaining energy*,* range (*r*), and velocity (*v*) **Output:** ScheduleList of registered CHs used by UAV for scheduling /*Initialize the network*/ 1:  **begin** prioritization process 2:   **for** (each CHs in the list) **do** /*Computation*/ //By using the information of size of packets, location coordinates, and remaining energy received in registration frame, UAV calculates the time CH remains in its coverage area. If CH has enough time and energy to communicate with UAV, then it is scheduled; otherwise, it is discarded. 3:     Calculates CDT and α: $CDT=\sqrt{\frac{\begin{array}{cc} r^{2}- & d^{2} \end{array}}{v^{2}}},$
*α* = CDT/*l*
//UAV uses this information for scheduling process 4:     **if** (α is the lowest among all registered CHs and the remaining energy is enough for communication) **then**
//UAV checks for the CH with the minimum value of *α* 5:       CH has the highest priority and is placed at first in the ScheduleList //UAV maintains a queue to keep the CHs in order of their priority 6:       **else if** (remaining energy of CH is not enough to complete communication with UAV) **then** 7:       CH is not scheduled and it is removed from the RegisteredCHList // From Algorithm 1 /*Update*/ //RegisteredCHList is updated **//**CH with the second lowest value of α is scheduled if it has enough energy for communication 8:     **end if** 9:    **end for** 10:  **end** /*Update*/ 11: Return ScheduleList

## 5. Analysis of Energy and Delay

We consider a UWSN consisting of *N* sensors within an area of interest *A,* where a UAV is employed as a mobile data collector to gather information from *n* cluster heads (CHs) on the ground. The location of *CH_i_* is (*x_i_*, *y_i_*). Each CH collects data packets from its cluster members. We assume that the UAV flies at a fixed altitude of *h* meters and its maximum speed is denoted as *S*_max_ in meters/second (m/s). The UAV’s path is pre-planned. Let *X_o_*and *X_f_*are the initial and final location of the UAV in the defined path. We assume $\left|\left|X_{f}-X_{o}\right|\right|\leq S_{\max}\times T$*,* where *T* is the data-gathering period of the UAV from CHs. CHs employ sleep and wake-up phases for energy consumption minimization. CHs wake up after sensing beacon signal from UAV. Only one CH can communicate with the UAV; otherwise, collision occurs. That is,

(5) $$P\;\left(UAV,CH\right)=\{\begin{array}{c} 1\\ 0 \end{array}\begin{array}{c} If\;connection\;between\;UAV\;and\;CH\;is\;established\\ Otherwise \end{array}$$

**Figure 4:**
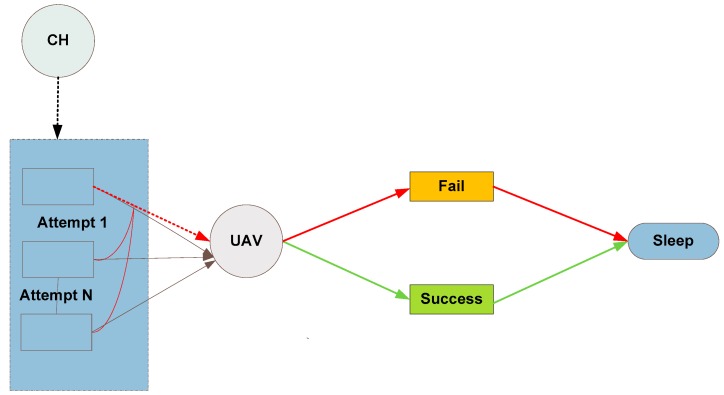
Communication process of EF-MAC.

The proposed UWSN model for EF-MAC is designed to minimize delay during UAV-to-CH communication without increasing the energy consumption of CHs. This is given by the following objective function:

(6) $$\mathrm{Minimize}\;\mathrm{delay}(D),\;\mathrm{Subject}\;\mathrm{to}\;E=E_{\min},$$

where *E*_min_ is the minimum possible energy required for UAV-to-CH communication. The communication process of EF-MAC is shown in Figure 4.

As shown in the figure, CHs try to communicate with the UAV to transfer data. If the CH senses the channel busy, it postpones its transmission by a random number that is back-off interval (0, *CW_i_-*1), *i* ∈ *n*, where *CW_i_* is the contention window size for particular CH. The value of contention window depends on the number of retransmissions attempts and is given as in [39]

(7) $$\;\;CWi=2^{j}CW_{\left(\min\right)},j\in \mathrm{number}\;\mathrm{of}\;\mathrm{attempts},$$

where the minimum value of *CW* is given by *CW*_min_ and *CW*_max_ gives the maximum value of back-off window. If the CH fails to communicate with the UAV within a number of attempts, data packets are discarded and CH sleeps to save the energy. We have considered certain retry limits so that energy waste due to long contention is minimized in our analysis. If CH is scheduled when it is in the active area, data is successfully transmitted to the UAV and CH sleeps.

**Figure 5:**
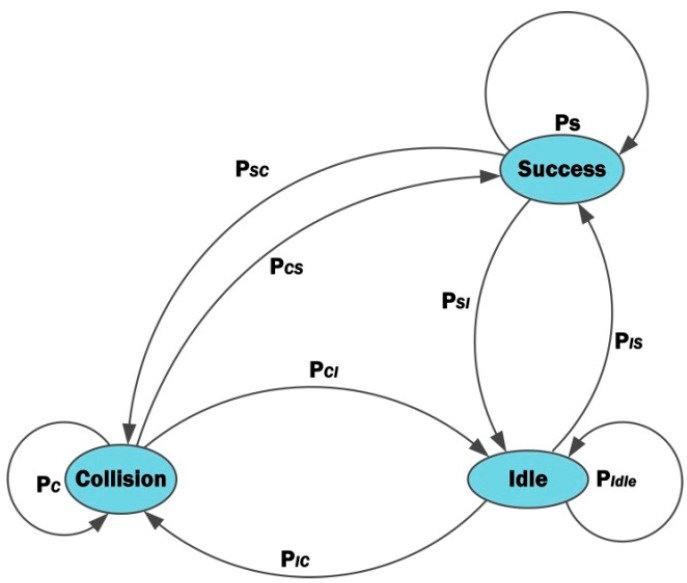
Three-state Markov model.

Figure 5 illustrates the Markov model for three different states (i.e., collision, success, and idle states) which basically define the energy and delay performance of the UAV-to-CH communication. CHs sleep in other time to save their energy. The back-off state of a CH is also included inside the idle state in the figure as the CH has to remain idle during the period. In a homogenous network, packet transmission probabilities are the same for all CHs. If *ϰ* is the probability, any CH has data packets to transmit, *ζ is* the probability to sense the channel, and *τ* is the probability to sense the channel busy, the probability for *CH_k_* to transmit at a given time *t* is given by:

(8) $$P_{t}(k|n_{c})=\overset{n_{c}}{\underset{i=1}{\prod }}\varkappa {\left(1-\tau \right)}^{\xi \;},$$

where *n_c_* is the number of contending CHs in particular time and region for *CH_k_*. Assuming that each transmission attempt is independent of previous ones, the probability that any CH (*CH_k_*), *k* ∈ *n_c_,* will transmit successfully in a given time is:

(9) $$P_{success}(k|n_{c})=\prod_{i=1,i\neq k}^{n_{c}}\left({\bar{P}}_{t\left(i\right)}\right)Pt(k),$$

where $\left({\bar{P}}_{t\left(i\right)}\right)$ is the probability that no CH will transmit in a given time. In case of multiple transmissions from CHs to UAV, collision occurs. The probability of collision for *CH_k_* is thus given by:

(10) $$Pcollision(k|n_{c})=\prod_{i=1}^{n_{c}-1}(Pt(i))$$

*CH_k_* changes its state either from success to idle state or from collision to back-off state. During its active period, the state of CHs is assumed to be any of the three states defined in the Marcov model. Hence, the sum of their steady-state probabilities is 1. Thus, the probability for *CH_k_*to be idle (either due to collision or after successful transmission) is:

(11) $$P_{idle}(k|n_{c})=1-(P_{collision}+P_{success})$$

If *m* is the number of simultaneous transmissions from different nodes, the state probabilities in Equations (9), (10), and (11) can be written as:

(12) $$P_{state}(m,p)=\{\begin{array}{c} P_{idle}\\ P_{success}\\ P_{collision} \end{array}\begin{array}{c} m=0\\ m=1\\ m>1 \end{array}.$$

If there is no any transmission or reception, then the node is said to be in idle state. If a single node is either transmitting or receiving, then it is in success state. If many nodes are transmitting or receiving at the same time, it is in collision state. If *t* is the present state, then state transition probabilities of next state (*t +* 1) are:

(13) $$\;\;P_{i\;}\left(t+1\right)=P_{i}P_{i\;}\left(t\right)+P_{ci}P_{c}\left(t\right)+P_{si}P_{s}\left(t\right),$$

(14) $$\;P_{c\;}\left(t+1\right)=P_{ic}P_{i\;}\left(t\right)+P_{c}P_{c}\left(t\right),$$

and

(15) $$P_{s\;}\left(t+1\right)=P_{is}P_{i\;}\left(t\right)+P_{s}P_{s}\left(t\right)$$

In Equation (13), *P_i_P_i_ (t)* gives the probability of a node continuing its idle state in the next state as well. *P_ci_P_c_* *(t)* represents the probability of a node to be in idle state after observing collision. *P_si_P_s_* *(t)* is the probability of node being in idle state after successful communication with the UAV. Similarly, in Equation (14), *P_ic_P_i_ (t)* gives the probability of a node to be in collision when it tries to communicate after its back-off time. *P_c_P_c_* *(t)* is the probability that nodes collide in the second attempt. There is no successful transmission in case of collision. Hence, the state transition from collision to success does not exist. In Equation (15), *P_is_P_i_ (t)* shows the transition of node from idle state to successful transmission state. *P_s_P_s_ (t)* is the probability that a node successfully communicates with the UAV for the second time. Moreover, in this equation, if a node succeeds to communicate with the UAV, the probability of switching to collision state is zero. From the above equations, the probability matrix of state transition is given by:

(16) $$P=\left[\begin{array}{ccc} P_{i} & {Pc}_{i} & P_{si}\\ \;P_{ic} & P_{c} & 0\\ P_{is} & 0 & P_{s} \end{array}\right]$$

If *E_Tot_*is the total energy consumed by any CH node, then:

(17) $$E_{Tot\;}=\sum_{i=1}^{3}P_{si\;}E_{si\;},$$

where *P_si_* is the probability of a node being in each of three sensors states, and *E_si_* is the energy dissipated by CH in corresponding states and is given by:

(18) $$E_{si\;}=E_{success\;}+E_{collision}+E_{idle},$$

where *E_success_* and *E_collision_* are represented as:

(19) $$E_{success\;}=E_{TX\;}+E_{RX\;}+E_{sense\;}$$

and

(20) $$E_{collision\;}=RE_{TX\;}+E_{sense\;},$$

where *E_Tx_*, *E_RX_, E_idle_,* and *E_sense_* are the energy consumed for transmitting, receiving, back-off stage, and sensing, respectively, and *R* is the number of re-attempts for CH to get registered.

Transmission energy depends on the packet size (*l*) and distance between the UAV and CH (*δ)* transmission energy and receiving energy is calculated as in [40,41]:

(21) $$E_{Tx}\left(l,\delta \right)=\{\begin{array}{c} l\times E_{elec}+l\times E_{amp}\times \delta^{2}\;\;\;\;\;if\;\delta <d^{\prime }\\ l\times E_{elec}+l\times E_{amp}\times \delta^{4}if\;\delta \geq d^{\prime } \end{array}$$

and

(22) $$E_{RX}=l\times E_{elec},$$

where *E_elec_* and *E_amp_* are the energy dissipated to run transmitter and the energy dissipated by the transmission amplifier with different distance level, respectively, and *d’* is given as:

(23) $$d^{\prime }=\sqrt{\frac{{\left(4\pi \right)}^{2}\times l\times R_{tr}\times R_{r}}{\lambda^{2}}},$$

where R_tr_ and R_r_ are the range of transmitter and receiver, respectively, and λ is the wavelength. The energy consumed by the *CH_k_* during three different states (success, collision and idle) is thus given by:

(24) $$E_{success(CHk)}=P_{success}(k|n_{c})\times [E_{sense}+(E_{Tx}\times l_{reg})+(E_{Rx}\times l_{sch})+(E_{Tx}\times l_{Tdata})]$$

(25) $$E_{colision(CHk)}=P_{collision}(k|n_{c})\times [(E_{sense}\times R)+(E_{Tx}\times l_{reg}\times R)]$$

and

(26) $$E_{idle({CH}_{k})}=P_{idle}(k|n_{c})\times (C_{Wk}+T_{idle})$$

where *l_reg_*_,_ *l_Tdata_*, *l_sch_*are the length of registration packet, the length of aggregated data packets by *CH_k_*, and the length of schedule packets, respectively. *T_idle_* is the time *CH_k_* remains in idle state. Then, the total energy depleted by *CH_k_* can be derived from Equations (24), (25), and (26) as:

(27) $$\begin{array}{ll} & E_{Tot({CH}_{k})}=E_{success({CH}_{k})}+E_{collision({CH}_{k})}+E_{idle({CH}_{k})}\\ = & P_{success}(k|n_{c})\times [E_{sense}+(E_{Tx}\times l_{reg})+(E_{Rx}\times l_{sch})+(E_{Tx}\times l_{Tdata})]+\\ & P_{collision}(k|n_{c})\times [(E_{sense}\times R)+(E_{Tx}\times l_{reg}\times R)+\\ & \left(E_{Rx}\times l_{sch})+(E_{Tx}\times l_{Tdata})]+P_{idle}(k|n_{c})\times ({CW}_{k}+T_{idle}\right)\\ = & P_{success}(k|n_{c})\times [E_{sense}+(l_{reg}\times E_{elec}+l_{reg}\times E_{amp}\times \delta^{2})+(E_{Rx}\times l_{sch})\\ + & \left(l_{Tdata}\times E_{elec}+l_{Tdata}\times E_{amp}\times \delta^{2})]+P_{collision}(k|n_{c})\times [(E_{sense}\times R\right)\\ + & (l_{reg}\times E_{elec}+l_{reg}\times E_{amp}\times \delta^{2})\times R]+P_{idle}(k|n_{c})\times ({CW}_{k}+T_{idle}). \end{array}$$

The delay observed by *CH_k_*can be calculated by dividing the process into two steps of registration phase (contention period) and data-gathering phase (contention-free). That is,

(28) $$T_{CSMA}=\sum_{j=1}^{R}D_{collision}\times 2^{j}CW_{\left(\min\right)}$$

and

(29) $$T_{TDMA}=D_{Queue}+D_{Trans}+D_{Prop}$$

where *D_collision_*is the delay due to collision while transmitting the registration frame which is given as:

(30) $$D_{collision}=(T_{slot(i)}+D_{p(i)}).$$

During contention-free phase, *CH_k_*simply uses assigned slot and transmits data. No any extra delay is expected due to collisions and retransmissions. Since variable-length TDMA slots are used, the transmitting time is different for CHs. The three different delays observed (i.e., *D_Trans,_ D_Queue_,* and *D_Prop_*) are calculated as:

(31) $$Transmission\;delay\;\left(\;D_{Trans}\right)=\sum_{i=1}^{lm}\frac{l_{Tdata(i)}}{BW},$$

(32) $$Queueing\;delay\;\left(D_{Queue}\right)=\sum_{j=1}^{PL}X_{j}+\omega ,$$

and

(33) $$Propagation\;delay\;\left(\;D_{Prop}\right)=\sum_{i=1}^{lm}\frac{\delta_{i}l_{Tdata(i)}}{BW}$$

respectively, where BW is the bandwidth of the channel used, *X_j_* is the service period of high priority nodes, ω is waiting time until it is scheduled, and *PL* is the number of priority levels for different CHs. The value of $l_{Tdata(i)}$ ranges between 100 and 150 bytes. If the steady-state vector is *v* = [*v_idle_ + v_c_ + v_s_*], the network throughput is:

(34) $$Throughput(Th)=\frac{\upsilon_{s\;}}{\sum_{i=1,i\varepsilon \left\{i,c,s\right\}}\upsilon_{i\;}}.$$

If *ψ* is the delay observed due to retransmissions of registration frames, then

(35) $$\psi =\frac{1}{R}\sum_{i=1}^{R}(CW_{i}/l_{reg})D_{Trans},$$

where *l_reg_* is the length of registration frame. If we consider the worst-case scenario, where *O* is the maximum offered slot size given as $O=\sum_{i=1}^{lm}P_{success}(k|nr)\times l_{Tdata(i)}$ and *CH_k_* is registered at the last retransmission attempt, the average packet delay experienced by *CH_k_* is:

(36) $$D=\left(\frac{O}{Th}-1\right)\psi +\frac{O\times \upsilon_{s\;}}{Th}\left(T_{CSMA}+T_{TDMA}\right)$$

**Figure 6:**
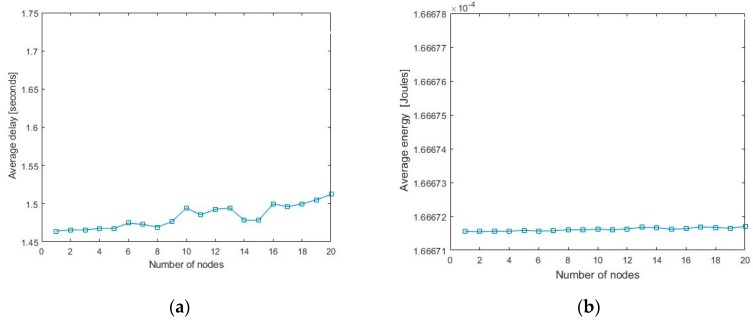
Results of the Marcov-based analytical model: (**a**) average delay experienced by the nodes and (**b**) average energy consumption of the nodes.

At the UAV side, the registration packets arrived from CHs are queued until the scheduling process begins. UAV immediately starts scheduling process once the queue is filled. It uses the second transceiver to share the scheduled information to the sensor nodes. Therefore, we assume that the delay incurred during the scheduling process is negligible. The numerical results of Marcov-based analytical model are shown in Figure 6, in which the average delay and average energy consumption for UAV-to-CH communication in the proposed scheme are shown. 

## 6. Performance Evaluation

In this section, the performance of the EF-MAC protocol is evaluated and compared with that of the two conventional protocols. For comparison with our work, we have selected the IEEE 802.11 CSMA/CA MAC first because it is extensively used for wireless communication in UWSNs. We have then selected the HP-MAC protocol because it is a recently proposed state-of-the-art ‘hybrid’ MAC protocol as in our EF-MAC. In our performance study, the MAC protocols under evaluation are simulated by using the Matlab R2018a simulator.

### 6.1. Simulation Environment

In our simulation, sensor nodes are randomly distributed in the area of 500 × 400 m, the UAV is assumed to fly at the height 10m and with the speed of 15 m/s. Out of the total sensor nodes deployed, 10 percent (10%) is supposed to be CHs, which are responsible for data transmission to the UAV. We have tested the results by increasing the number of sensor nodes. For the proficient operation of the protocol, we have chosen constant height and velocity of the UAV such that every CH will get an access to the UAV at least once during the flight time.

We have considered key metrics to evaluate the performance of the proposed MAC and to compare it with existing ones. The performance metrics are as follows:

- Network latency: As we have targeted our work for disastrous and emergency situations, delay is primarily focused. However, we have narrowed our work to the average time consumed for UAV-to-CHs communication. Total network delay considered in our UWSN network is given in Equation (36).
- Fairness: In every resource sharing system, all the users should necessarily be treated equally such that network would perform fairly. Fairness is a key point under high mobility context as in UWSNs where every sensor should communicate in limited time for better utilization of the network. It is the function of variability of throughput to the users. For any set of user throughputs $\left(x_{1},x_{2},x_{3},\ldots x_{n}\right),$ the fairness index of a network can be assigned as:
  (37) $$f\left(x_{1},x_{2},x_{3},\ldots x_{n}\right)=\frac{{\left(\sum_{i=1}^{n}x_{i}\right)}^{2}}{n\sum_{i=1}^{n}{\left(x_{i}\right)}^{2}}$$
- For all non-negative values of *x_i_’s*, the fairness index lies between 0 and 1. In the proposed UWSN network, if all the sensor nodes get the equal chance of communicating with the UAV, then fairness is indexed as one. Sensor nodes may have different duration to be in contact with the UAV and the size of data to be transmitted varies. Therefore, the weighted fairness regarding the CDT and packet size to be transmitted is required when evaluating the fairness in our prioritization algorithm. For any *CH_i_*, i.e., *n*, the weighted fairness index is the ratio of CDT of that particular CH to the time required for transmitting data by that CH (*W_fi_ = CDT_i_/T_i_*). Thus, the opportunity for every sensor to participate in communication process is given by
  (38) $$Fc=\frac{{\left(\;\sum_{i=1}^{n}N_{slots}(i)\times W_{fi}\right)}^{2}}{n\sum_{i=1}^{n}{\left(N_{slots}(i)\times W_{fi}\right)}^{2}},$$
  where *N_slots (i)_* is the size of time slot allocated to CHs that is of variable length. If the UAV spends much time in gathering data from certain sensors only then it may not have enough time to address all the sensors.
- Energy efficiency of sensor nodes: Energy efficiency of UWSN network is defined as the amount of energy consumed by the wireless sensor nodes during the communication with UAV. Regardless of the number of sensor nodes used in UWSN network, CHs are responsible to aggregate the received information from the cluster members to l bits and transmit it to UAV. The major reason of energy exhaustion in sensor nodes is due to its radioactivity i.e., transmission and reception of data. Hence, much works are being carried out to minimize the number transmission and reception as a result of collisions. We have assumed the distance between CH and the UAV as *δ*, and then the energy consumption is calculated as shown in Equation (27).
- Throughput: It is the rate of data delivery at the MAC layer of the network. In other words, it is the total amount of data transmitted to the UAV by the sensors. Let, *Φ* denotes average traffic that is generated in the observed area per unit time. Total traffic per distance *δ* is *Φδ*. If *S_(x)_* is the probability that a packet is successfully delivered to the UAV at *δ* i.e., *S_(x)_* = (1*−P^Txm^*) where *P* is the probability of packet failure and *Txm* is the maximum attempts made. Then, the overall throughput of the network is given as expressed in seconds and evaluated as:
  (39) $$Throughput(Th)=\int_{0}^{A}\Phi \delta S_{\left(x\right)}dx$$
- Network lifetime: The operation of UWSN is subject to multiple constraints, among which one of the most critical is the available energy of the nodes in UWSN. Network lifetime is an important parameter in UWSN networks, which shows the number of dead nodes in every round of simulation due to the exhaustion of energy. Sensor nodes commonly use lithium thionyl chloride battery (*L_i_/SOCL_2_*) which has the capacity of 400–35000 mA. The lifetime of the network can be expressed in seconds and evaluated as:
  (40) $$lifetime=\frac{{energy}_{initial}}{{energy}_{total}},$$
  where *energy_initial_* is the energy of sensor node during its deployment and *energy_total_* is the amount of total energy depleted during transmission, reception and other radio activities and expressed as ${energy}_{total}=E_{Tx}\left(l,d\right)+E_{Rx}\left(l\right)$.
- Runtime complexity: The asymptotic computational complexity of the proposed algorithm is estimated on the basis of the instructions provided in the algorithm. The number of basic linear operations is calculated as *O*(*n*), where *n* is the number of clusters. In our algorithm, we have used a RegisteredCHList and ScheduleList to keep the records of CHs. The complexity of inserting elements is 2×*O*(log *n*). The proposed scheme is accompanied by *m* transmissions from *n* clusters. Its complexity is given by *O*(*mn*). Hence, the asymptotic complexity of the algorithm is *O*(*n*) *+* 2×*O*(log *n*) *+ O*(*mn*) *= O*(*mn*).

Simulation parameters are listed in the Table 1.

### 6.2. Simulation Results and Discussion

**Figure 7:**
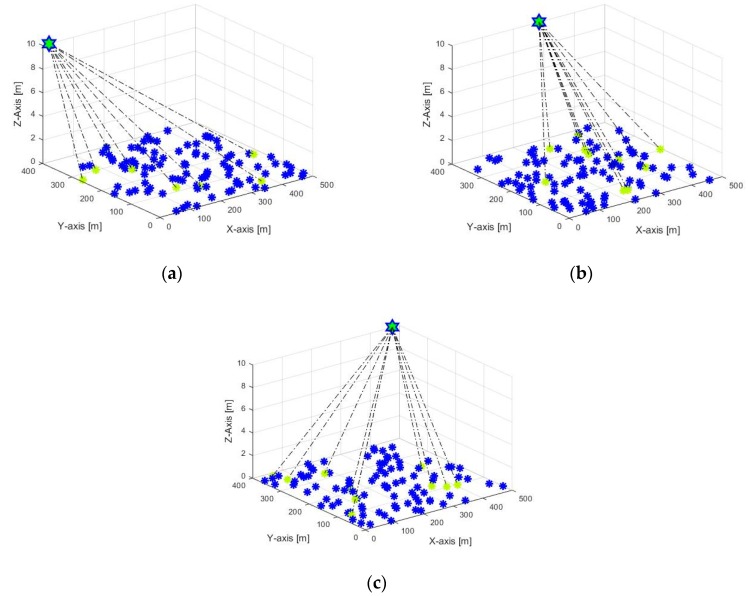
UAV based data collection in WSN with three different antenna orientations of (**a**–**c**).

In this section, results obtained via simulation are summarized and then they are compared with other conventional MAC protocols for various parameters mentioned in the above section. The simulation results in Figure 7 depict the implementation of the UAV in WSN environment for data gathering process with different antenna orientation. Implementation is done in 3D scenario. We assume that the UAV uses a simple trajectory which is defined and controlled by the BS. Because our work aims to enhance the CH-to-UAV communication, we consider the mobility and height of the UAV within the WSN deployment region only. For simplicity, it is assumed that the UAV moves with constant speed and at fixed height as in Figure 7. In the simulation, the UAV receives the information about CHs and their location while it is moving. That is, the UAV’s path covers all the sensors while it is moving within the WSN area. The UAV keeps gathering data from CHs while it is moving without stopping or slowing down at any point.

**Figure 8:**
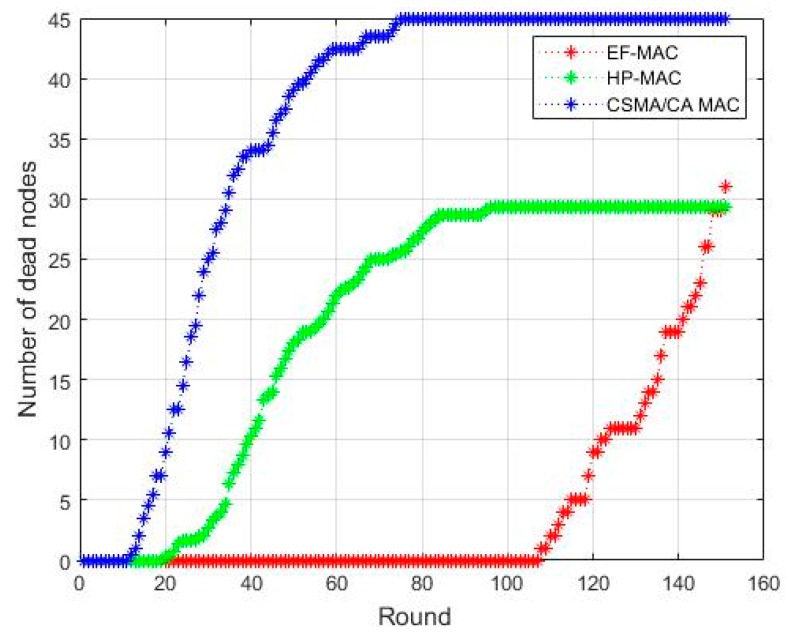
Network Lifetime.

Figure 8 shows the lifetime of the proposed EF-MAC network. Simulation is done for the number of rounds to check the number of dead nodes, which is due to the depletion of energy. It is very apparent from the figure that the number of dead nodes is proportionally high for CSMA/CA MAC than other two MAC protocols. It is due to the number of collisions and re-transmissions during data transmission process. Furthermore, nodes need to stay active for longer duration contending the channel in CSMA/CA. This significantly affects the energy efficiency of the network. Number of dead nodes for HP-MAC is comparatively lesser than that of CSMA/CA but higher than that of the EF-MAC due to the fact that in the proposed EF-MAC nodes need not to stay active for long time to send data and also the channel is very efficiently used.

**Figure 9:**
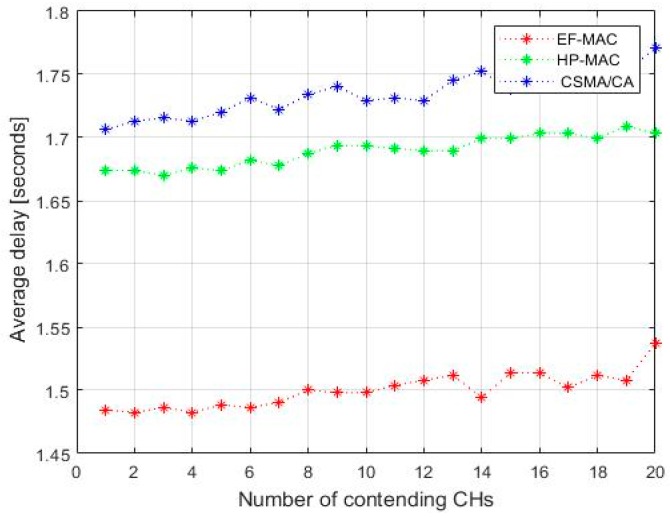
Network Latency.

Figure 9 shows the time taken for a sensor node (CH in EF-MAC) to communicate with UAV. In addition, we have checked the result by varying the number of contending nodes. It can be observed that there is certain delay in the network as the number of nodes is increased. However, there is not significant increment in the time duration (only 0.05) even when the number of nodes is increased by five times. Thus, the result shows that the proposed system performs well even in the highly dense network. Afterwards, the result is compared with two other MAC protocols i.e., CSMA/ca MAC and hybrid HP-MAC. The result of comparison shows that the delay is reduced in the proposed EF-MAC by 20% and 25% than in HP-MAC and CSMA/CA MAC. This is due to the fact that both of the transceivers are not used for data-gathering. The time for broadcasting beacons and receiving beacons is minimized which is much shorter than the time for scheduling and data-gathering process. The time slots are assigned and used efficiently which also minimizes the network delay. Moreover, the data are aggregated by the CH and only useful information is sent to the UAV using LOS communication.

**Figure 10:**
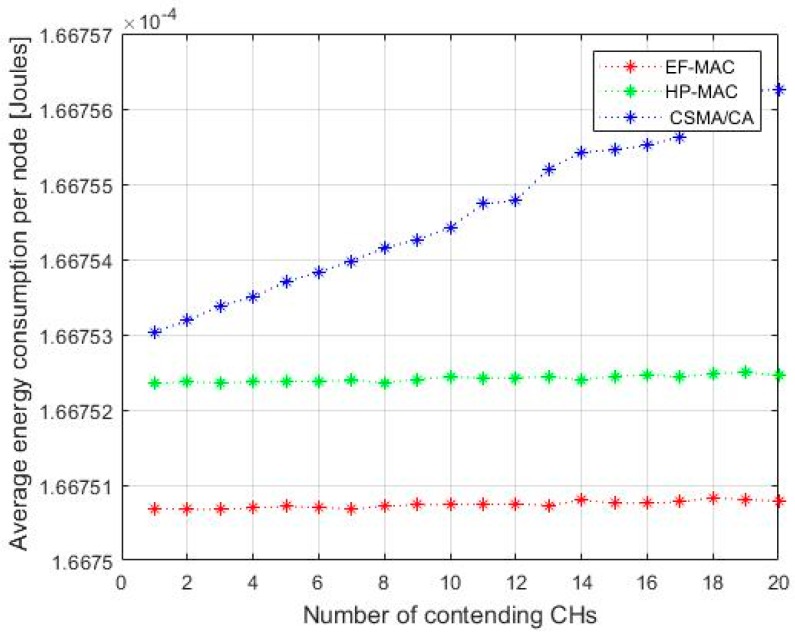
Average energy consumption.

From the results obtained in Figure 10, we can say that the proposed EF-MAC reduces the energy consumption than the previously proposed MAC protocols i.e., CSMA/CA MAC and HP-MAC. Reduction in energy is due to the minimum time for sensors to remain active and communicate with UAV, a smaller number of collisions, re-transmissions, and variable slot-time TDMA slots which restricts the wastage of time during the data transmission process. Furthermore, the nodes are clustered and only CH takes part in UAV-to-CH communication, which also minimizes the energy depletion. As there is proper handshaking between the UAV and CHs before data transmission process, there is nominal chance of idle listening and overhearing. Energy consumption is thus reduced by 12% than in HP-MAC and by 17% than in CSMA/CA MAC protocol in EF-MAC.

**Figure 11:**
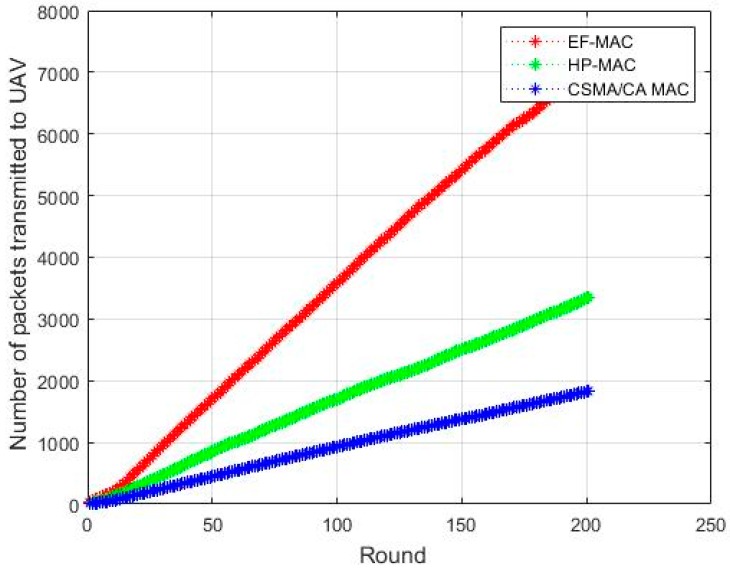
Packet delivery per rounds.

Figure 11 shows the total number of data packets transmitted to the UAV from CHs within the different rounds of data gathering phase. It can be figured out that the number of packets transmitted is highest in the proposed EF-MAC than other two conventional MAC protocols. In EF-MAC, UAV can receive data using two channels and the information about the number of data packets is used by UAV during scheduling process. This helps the UAV to assign slot time properly such that more number of packets can be received in the given time. There are minimal chances of packet loss due to the coverage problem and the lifetime of sensor nodes in EF-MAC is improved one. Ultimately, the number of nodes communicating is increased and so is the number of packets collected by UAV. Hence, the throughput of the EF-MAC is also improved and the base station can take right decision with the sufficient information received from the sensor nodes.

**Figure 12:**
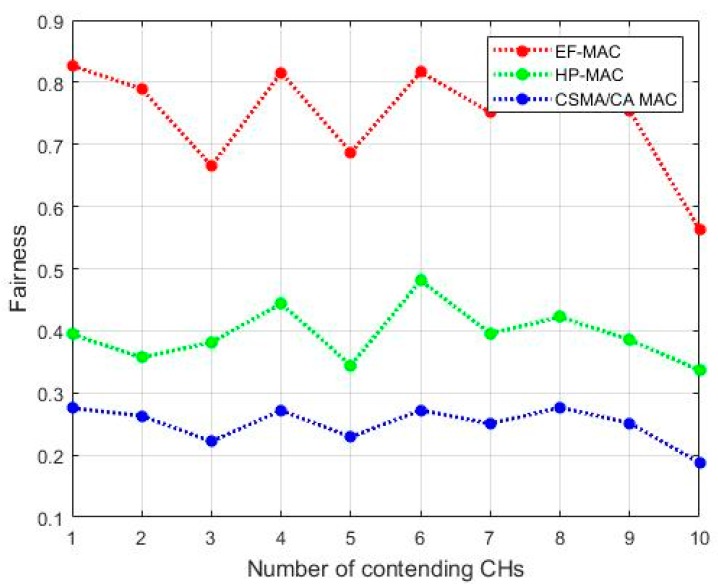
Fairness.

Figure 12 depicts the simulated fairness of EF-MAC for various number of contending nodes. In the proposed UWSN system, the network is supposed to be fair enough if every CH gets equal chance of communicating with UAV and transmitting its data. In this scenario, EF-MAC has improved fairness than HP-MAC and CSMA/CA MAC. EF-MAC uses prioritization method, which uses information such as duration CH remains in UAV’s coverage area, packet size and remaining energy to assign priority such that every CH has chance of participating in the communication process before it dies or gets out of the active region. Fairness level is indexed very low in case of CSMA because there is high chance that many CHs get out of the active region of the UAV before transmitting its data to the UAV or due to the collisions occurred while transmitting data packets. Meanwhile, in the proposed EF-MAC two transceivers are concurrently operated which escalates the probability for every CH to participate in communication process. However, the result shows fluctuating values of fairness, which is because of the variable size of, time slots assigned as in Equation (38). If data is collected from every CH then the network is also assumed reliable one.

## 7. Conclusions

In this paper, we have presented an energy-efficient and fast MAC protocol called EF-MAC for time-critical applications such as border surveillance, pre- and post-war environments and disaster-prone atmospheres. Reliable and timely communication in case of surveillance and aftermath of natural calamities helps to minimize the loss of lives and properties to the great extent. If the information of unauthorized border crossing by trespassers, smugglers, and terrorists is transmitted to the base station without much delay, then the instant action could be taken against it. In addition, during wars and disasters, proper rescue can be arranged, which saves lives of many people. To address these issues, variable slot-time TDMA that reduces the unnecessary energy and time of the network has been presented in this paper.

The proposed EF-MAC adaptively handles the varying amount of data traffic by using a large number of small size data slots. EF-MAC has been analytically evaluated using the Markov model and then validated through the extensive simulation. The simulation results closely resemble the results obtained through the Marcov-based analytical model in terms of energy consumption and time. From the performance study, it is apparent that the time to communicate with the UAV for sensors is highly reduced and the energy consumption is lessened. Thus, we can target our protocol for the critical environment where network latency is primarily concerned.

The use of multiple UAVs could be considered rather than a single UAV for this work, but the coordination and efficient communication between UAVs must be guaranteed for better performance of the protocol. In addition, designing an efficient path-planning algorithm and synchronizing the UAV’s arrival time with the active period of CHs can be thought-out so that energy wasted during the periodic wake up can be minimized. We have left this as our future work. As another future work, we will study the optimality of MAC protocols in UWSNs in terms of energy consumption and network latency.

## Figures and Tables

**Table 1:** Simulation parameters.

Parameter	Value
Network simulator	Matlab
UWSN deployment area	500 m × 400 m × 10 m
Number of sensor nodes	100–200
Number of UAV	1
Initial energy	2 J
Transmission energy	17.1 nJ
Receiving energy	15 nJ
Idle state energy	12.7 nJ
Sensing energy	2.7 nJ
Packet size (variable)	100–150 bytes
Registration frame size	20 bytes
Maximum queueing delay	20 × 16 *×* 10^−6^ s
Back-off value	rand (3, 5)
UAV’s speed	15 m/s
AV’s height	10 m
UAV’s transmission range	200–250 m
Sensors	Static
Directional antenna flare angle	60°
